# Successful biosynthesis of natural antioxidant ergothioneine in *Saccharomyces cerevisiae* required only two genes from *Grifola frondosa*

**DOI:** 10.1186/s12934-020-01421-1

**Published:** 2020-08-18

**Authors:** Ying-Hao Yu, Hong-Yu Pan, Li-Qiong Guo, Jun-Fang Lin, Han-Lu Liao, Hao-Ying Li

**Affiliations:** 1 Department of Bioengineering, College of Food Science and Institute of Food Biotechnology, South China Agricultural University, Guangzhou, 510640 China; 2 Research Center for Micro-Ecological Agent Engineering and Technology of Guangdong Province, Guangzhou, 510640 China

**Keywords:** Ergothioneine, *S. cerevisiae* cell factory, *Grifola frondosa*, Heterologous expression

## Abstract

**Background:**

Ergothioneine (EGT) has a unique antioxidant ability and diverse beneficial effects on human health. But the content of EGT is very low in its natural producing organisms such as *Mycobacterium smegmatis* and mushrooms. Therefore, it is necessary to highly efficient heterologous production of EGT in food-grade yeasts such as *Saccharomyces cerevisiae*.

**Results:**

Two EGT biosynthetic genes were cloned from the mushroom *Grifola frondosa* and successfully heterologously expressed in *Saccharomyces cerevisiae* EC1118 strain in this study. By optimization of the fermentation conditions of the engineered strain *S. cerevisiae* EC1118, the 11.80 mg/L of EGT production was obtained. With daily addition of 1% glycerol to the culture medium in the fermentation process, the EGT production of the engineered strain *S. cerevisiae* EC1118 can reach up to 20.61 mg/L.

**Conclusion:**

A successful EGT de novo biosynthetic system of *S. cerevisiae* containing only two genes from mushroom *Grifola frondosa* was developed in this study. This system provides promising prospects for the large scales production of EGT for human health.

## Background

Ergothioneine (EGT) is a derivative of hercynine with a sulfhydryl group attached to the second carbon atom on the imidazole ring and in the form of thione at pH 7. The EGT reduction potential energy is − 60 mV, which is higher than that (− 200 mV to − 320 mV) of other thiol structure antioxidant substances (such as glutathione and cysteine) and can effectively reduce auto-oxidation [1]. The situation showed that part of the product formed by the oxidation of EGT can be recombined back to EGT without a reducing agent, and the by-product contains sulfurous acid, which can be used as another reducing agent to counteract the intracellular oxidation state [2]. According to Cheah et al. [3], EGT accumulates at quite high levels in human tissues and cells, such as bone marrow, liver, kidney, semen, eyes and red blood cells. A group of investigators proposed that EGT may help relieve some of the symptoms associated with Cardiovascular disease [4], Crohn disease [5], and Alzheimer disease [6]. Cheah et al. [7] demonstrated that through oral administration of EGT, biomarkers of inflammation decreased and EGT was consider as a potential inhibitor for inflammation-related DNA halogenation [8]. Furthermore, EGT also reveals protective effect against hyperglycaemia-induced senescence [9] and smoke-induced oxidative damage [10]. Besides, some human cells, such as corneal endothelial [11], dermal fibroblasts [12] and keratinocytes [13] can be protected by EGT from oxidative attack. Additionally, EGT can chelate with Cu^1+^ [14] or Cu^2+^ [15] to be inert that prevent DNA and proteins form metal ions damage.

As a matter of fact, EGT cannot be synthesized in plants or animals and can only be synthesized by some bacteria and filamentous fungi, such as *Mycobacterium smegmatis* [16], Cyanobacteria [17], *Neurospora crassa* [18], and many mushrooms [19–21]. However, the EGT contents in original hosts are very low (range from 0 to 116 mg/100 g of dry weight in many species counting from Cumming et al. [22]) and the requirement for complex extraction or purification procedures of EGT from original producing organisms, restricting the commercially feasible in EGT production. There is need to develop a highly and efficiently method to increase EGT production, such as overexpressing the heterologous EGT biosynthetic genes in *Saccharomyces cerevisiae*. Moreover, *S. cerevisiae* is a safe host (GRAS) which can be used in food additives and cosmetics industries [23].

**Fig. 1:**
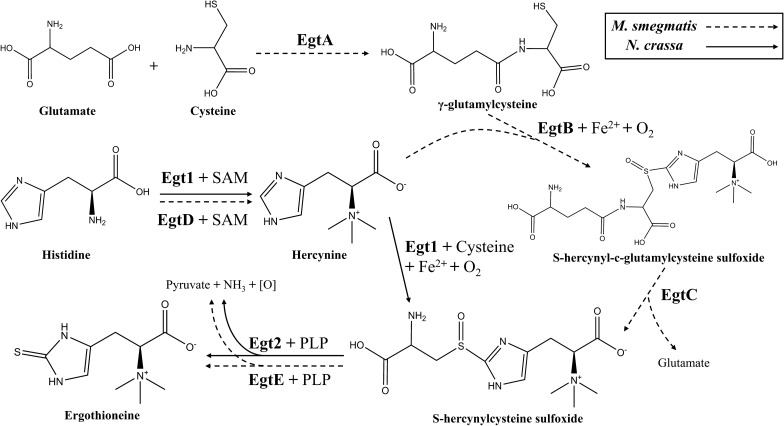
Two different pathways for EGT biosynthesis

To date, two distinct biosynthetic pathways of EGT have been extensively studied (Fig. 1). One of them was first discovered in *M. smegmatis* containing the gene cluster *egtABCDE* [24], also similar in *Methylobacterium* species [25] and *Streptomyces* species [26]. On the other side, Hu et al. [27] firstly identified the EGT biosynthetic genes *egt1* and *egt2* which was homologous to *egtBD* and *egtE* respectively in the filamentous fungi *N. crassa*. Soon after, homologous for the genes *egt1* and *egt2* were also found in *Schizosaccharomyces pombe* [28]. *Grifola frondosa* is a kind of mushrooms that contains a variety of active substances and the contents of EGT are 0.29–1.11 mg/g (dry weight) [19, 20] which are higher than many other species reported. It is worth investigating the function of EGT biosynthetic genes in *G. frondosa* through heterologous expression system. *S. cerevisiae* is a eukaryotic expression host providing many kinds of modifications by organelles and is commonly used for natural products biosynthesis [29]. Therefore, heterologous expressing EGT biosynthetic genes from *G. frondosa* in *S. cerevisiae* could be a practicable way to produce EGT in large scale.

In this study, the genes associated with EGT biosynthesis in the genome database of *G. frondosa* were analysed, and homologous genes were identified. We used RT-PCR to clone these genes and then overexpressed these two genes through pRS42K in *S. cerevisiae*. The results determined that heterologous overexpression in EGT synthesis only requested two genes from *G. frondosa*, which was simpler than five genes from *Mycobacterium smegmatis* [30] and three genes from *Flammulina velutipes* [31]. Higher EGT content was achieved to 20.61 mg/L by adjusting the carbon source and daily supply of glycerol in 168 h fermentation. Overall, de novo biosynthesis of EGT from glycerol in *S. cerevisiae* is a cost-effective and scalable strategy in commercial compared to chemosynthesis or extracting from other original organisms producing EGT.

## Results

### Genes cloned from *Grifola frondosa*

To clone the EGT biosynthetic genes from *G. frondosa*, the BLAST tool (https://blast.ncbi.nlm.nih.gov/Blast.cgito) was used to identify the corresponding sequence. Based on the gene sequences of *N. crassa egt1* (NCU04343) and *egt2* (NCU11365), we predicted that three hypothetical proteins, A0H81_08706 (with the *S*-adenosyl-l-methionine (SAM)-dependent methyltransferase domain), A0H81_08707 (with the 5-histidylcysteine sulfoxide synthase domain) and A0H81_07972 (with the pyridoxal phosphate (PLP)-dependent cysteine desulfurase domain), were involved in the EGT biosynthetic pathway. The *Gfegt1* sequence (Additional file 1: Table S6) cloned by *Gfegt1*-F and *Gfegt1*-R was 2580 bp, and its protein consisted of 859 amino acids with a 34% homology to NcEgt1. Similarly, the *Gfegt2* sequence (Additional file 1: Table S6) cloned by *Gfegt2*-F and *Gfegt2*-R was 1434 bp, and its protein consisted of 477 amino acids with a 33% identity to NcEgt2.

### EGT production in recombinant *S. cerevisiae*

**Fig. 2:**
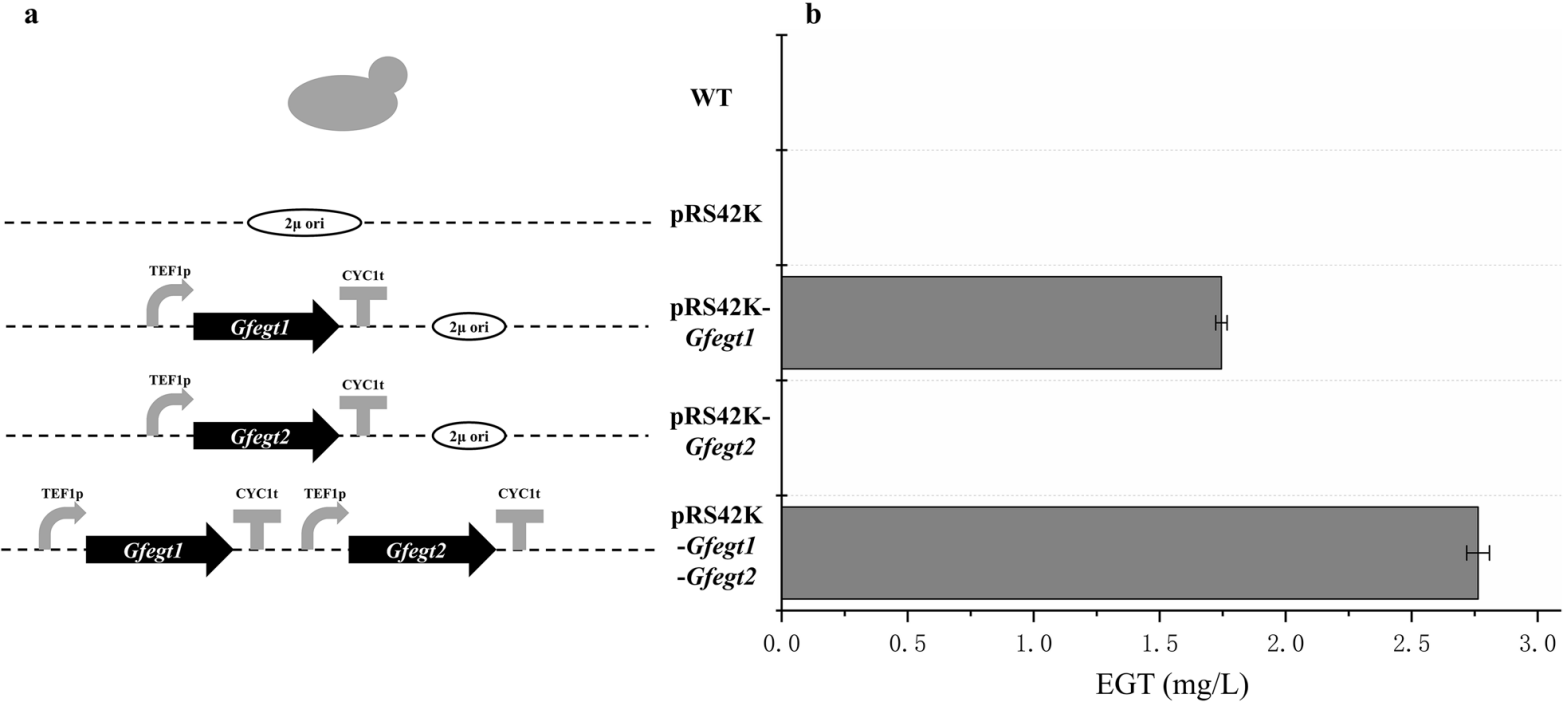
EGT biosynthesis in *S. cerevisiae* containing different construction plasmids. **a** Different construction types of strains. **b** EGT extraction contents from different types of strains

**Fig. 3:**
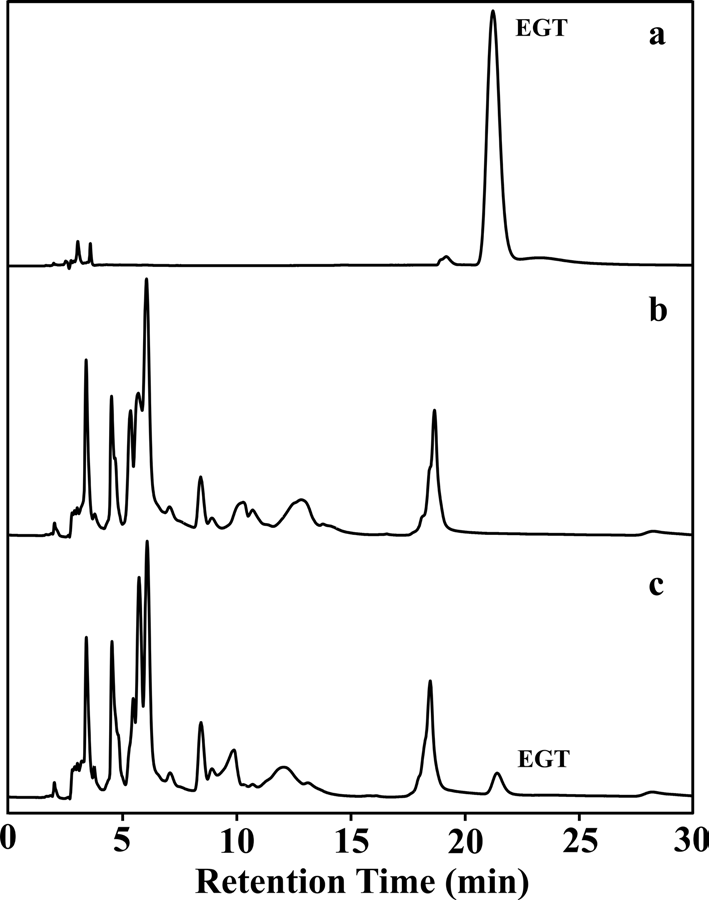
HPLC separation and identification of EGT extracting from cells. **a** EGT Standards. **b** *S. cerevisiae* EC1118. **c** *S. cerevisiae* EC1118 carrying plasmid pRS42K-*Gfegt1*-*Gfegt2*

To examine the relative enzyme activity of GfEgt1 and GfEgt2, four recombinant yeasts (Fig. 2a) were constructed with plasmids pRS42K, pRS42K-*Gfegt1*, pRS42K-*Gfegt2*, and pRS42K-*Gfegt1*-*Gfegt2*, respectively. The EGT content appeared only in cell extraction and cannot be detected in culture medium. The HPLC results (Fig. 3) showed that the retention time of the EGT standards was approximately 21 min, and an extraction sample of strain expressing both GfEgt1 and GfEgt2 proteins displayed a corresponding peak at the same time, while the WT sample peak was absent. In the present study, we found that the strain expressing only GfEgt1 contained 1.74 ± 0.02 mg/L EGT, while no EGT was detected in the strain expressing only GfEgt2 (Fig. 2b; Additional file 1: Table S1). In addition, the strain with pRS42K-*Gfegt1*-*Gfegt2* exhibited the ability to produce up to 2.76 ± 0.04 mg/L EGT which was 57% higher content than the strain expressing only GfEgt1. As a result, we consider that GfEgt1 and GfEgt2 are the key proteins for biosynthesizing EGT in *G. frondosa*.

### Identification of EGT

**Fig. 4:**
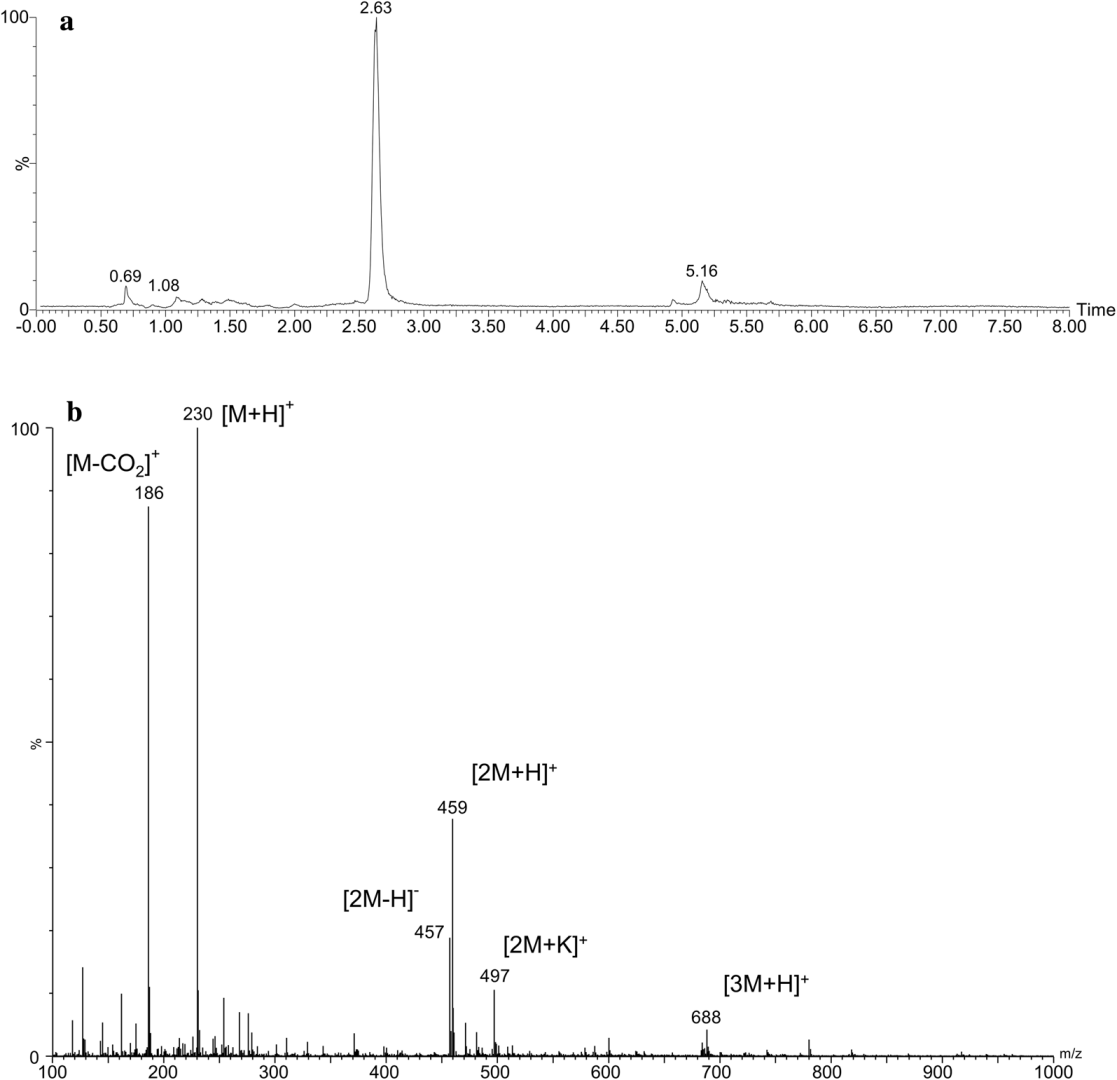
UPLC-ESI–MS analysis of EGT extraction from recombinant strain. **a** Chromatogram of EGT monitored at 230 m/z. **b** Electrospray ionization mass spectrum of EGT obtained from extraction

The UPLC chromatogram monitored at 262.7 nm and TOF MS ES+-selected ion monitored at m/z 230 confirmed that EGT was eluted at 2.63 min (Fig. 4a). The results of ESI–MS revealed a main molecular ion [M+H]^+^ of m/z 230.0971, which was consistent with the calculated exact molecular ion mass of 230.0963 (PPM = 3.5). Identification of the fragment ions were as follows: m/z 186 for 2-(2-mercapto-imidazol-5-yl)-N,N,N-trimethylethan-1-aminium (C_8_H_16_N_3_S^+^), which is the molecule of EGT lacking carboxyl group; m/z 497 for the sodium cluster ion [2M+K]^+^; and m/z 467, 459 and 688 for the cluster ions [2M−H]^−^, [2M+H]^+^ and [3M+H]^+,^ respectively (Fig. 4b).

### Carbon source of fermentation

**Fig. 5:**
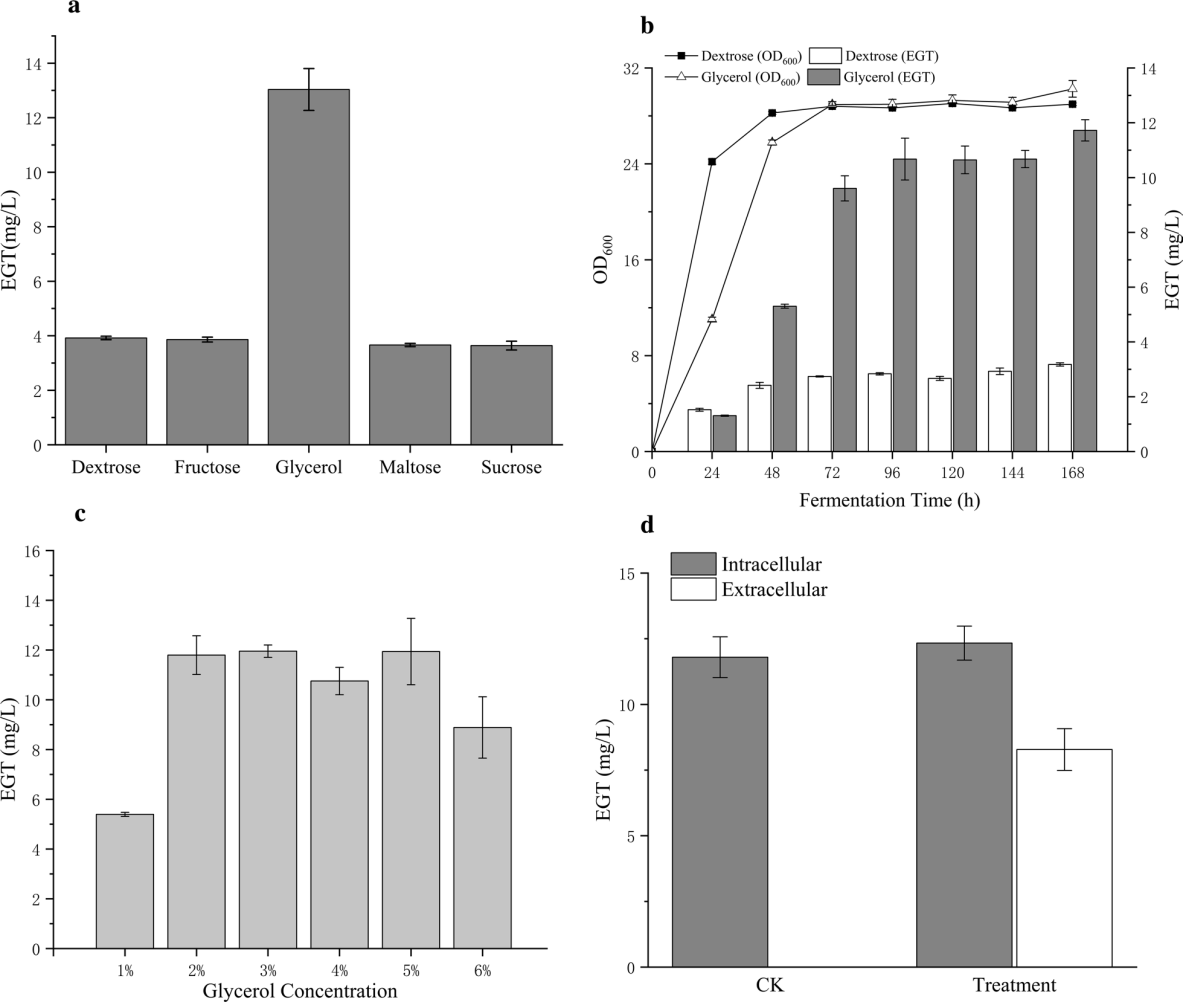
Optimization in EGT biosynthesis in *S. cerevisiae* with plasmid pRS42K-*Gfegt1*-*Gfegt2*. **a** Comparison of different carbon sources for EGT biosynthesis. **b** EGT contents and yeasts growth in each fermentation day. **c** Comparison of different glycerol concentration for EGT biosynthesis. **d** EGT biosynthesis through adding 1% glycerol in each fermentation day based on YPD medium (Treatment) and only based on YPD medium (CK)

Amino acids are the important reaction substances in the biosynthesis of EGT. Histidine is the starting material for the entire synthetic pathway. Cysteine provides sulfur atoms to EGT. Methionine is a precursor of SAM which is a source of methyl reactions. Different carbon sources lead to different amino acid contents and five different carbon sources (dextrose, fructose, glycerol, maltose, and sucrose) were used for the growth and amino acid production of yeasts in our study. We found that the EGT production from glycerol was 3.3-fold higher than that from dextrose (Fig. 5a; Additional file 1: Table S2). Subsequently, we analysed the fermentation conditions of dextrose and glycerol as carbon sources in each fermentation day (Fig. 5b; Additional file 1: Table S4). After activating the strains by YPD, we separated them into different carbon sources to ferment. The yeasts grown on dextrose displayed good vitality, while those grown on glycerol exhibited delayed cellular growth. However, both growths were in a plateau phase on the 3rd day and the EGT in vivo from glycerol exceeded that from dextrose at the 2nd day and reached a high level of approximately 10.68 ± 0.76 (mg/L) at the 4th day.

In addition, different concentrations of glycerol were used to ferment the recombinant strain with pRS42K-*Gfegt1*-*Gfegt2* (Fig. 5c; Additional file 1: Table S3). The analysing samples were mixed with intracellular and extracellular production. The results showed that fermentation using 1% glycerol as the only carbon source produced 5.39 ± 0.08 mg/L EGT, while 2–6% glycerol as the carbon source demonstrated a high yield of EGT, which was twofold higher than the former. However, there was no significant difference between the groups using 2% to 6% glycerol as the carbon source for fermentation. The higher concentration resulted in the inhibition of EGT synthesis. Considering the cost-effectiveness in manufacturing, 2% glycerol as the carbon source is a positive choice. Accordingly, the addition of all carbon sources at once was not feasible. We used YPG (2% glycerol) as the fermentation broth and added 1% glycerol each day for 7 days of fermentation. The result showed that the intracellular and extracellular EGT contents of treatment sample were 12.33 ± 0.65 mg/L and 8.28 ± 0.80 mg/L respectively, while EGT could not be detected from the extracellular in control sample. The total EGT content in treatment sample was about 20.61 mg/L (Fig. 5d; Additional file 1: Table S5). This phenomenon indicated that the suitable utilization of glycerol was a continuous progress.

## Discussion

In this research, we studied and compared the genomic database of the mushroom *G. frondosa* using the *N. crassa* EGT synthase NcEgt1 and NcEgt2 and found that there were three unidentified proteins: the unidentified protein A0H81_08706 with medium homology to the first half of NcEgt1, the unidentified protein A0H81_08707 with medium homology to the latter half of NcEgt1, and the unidentified protein A0H81_07972 with medium homology to NcEgt2. Firstly, we speculated that these three proteins were involved in the EGT synthetic pathway in *G. frondosa*. Then, the target fragments for these three proteins were cloned. However, the amplified sequence for A0H81_08706 was not a complete ORF because there was one more base at position 937 in the gene sequence, which caused a subsequent frameshift mutation. Thus, The *G. frondosa* genome was extracted and used as a template to amplify the genes. It was found that this frameshift also existed in the genome, excluding the mutation that occurred in the process of transcription. Since the proteins A0H81_08706 and A0H81_08707 were adjacent, we regarded them as one protein and designed primers to subsequently clone a complete ORF. NcEgt1 from *N. crassa* possesses two domains which one is the same as EgtD and another is the same as EgtB in bacteria for the EGT synthetic enzymes, corresponding to proteins A0H81_08706 and A0H81_08707, respectively, in *G. frondosa*. However, the gene cloned from our laboratory strain had just one complete ORF containing two domains. It can be inferred that with the evolution of species and the gradually diversifying in modification function of the protein, the combination of these two enzymes to form only one enzyme contributes to the simplification of the synthesis step. Additionally, the sulfoxide synthase domain of NcEgt1 has been verified [27], but there was no methyltransferase activity research in the methyltransferase domain of NcEgt1. It is worthwhile to investigate whether the methyltransferase domain of GfEgt1 is functional or not in our coming research.

According to the results, with the different combined expression of EGT synthase, there was only GfEgt2 expression without EGT production. On the contrary, in the presence of only the GfEgt1 expression, EGT was produced. These results indicated that *S. cerevisiae* might have a protein with similar functions as that of Egt2 and lacked an enzyme that functions similarly to Egt1. Using NcEgt1, NcEgt2 and egtB, egtD, egtE as the source alignment, we searched the *S. cerevisiae* gene and protein database and found no genes or proteins homologous to these proteins. Comparing the two genes, SPBC1604.01 (homologous to NcEgt1) and SPBC660.12c (homologous to NcEgt2), which were related to the biosynthesis of EGT in *S. Pombe* [28], we found no results of SPBC1604.01, but there was a kynureninase catalysing transamination reaction in *S. cerevisiae* discovered by aligning it with SPBC660.12c. This enzyme consists part of cysteine desulfurase (SufS) domain and it is possible that its desulfurization function is not found yet. Similar to our results, only heterogenous expression of NcEgt1 in *Aspergillus oryzae* can also synthesize EGT [32]. Besides, Irani et al. [33] proposed the cleavage of the C-S was a main reaction between S-hercynylcysteine sulfoxide and PLP. It is predictable that two substances can react directly before binding to NcEgt1 or GfEgt1.

The reaction substrates and coenzymes participating in the EGT synthetic pathway can be synthesized by *S. cerevisiae* in vivo. SAM, the methyl donor for hercynine, was synthesized by *S*-adenosyl-l-methionine synthetase (EC 2.5.1.6) from methionine. The coenzyme of NcEgt2, PLP, is the active form of vitamin B6, produced from glycolysis and the pentose phosphate pathway. We used *S. cerevisiae* to express two EGT synthetases, and the fermentation by basic medium was free from additional enzymatic reaction materials. In our research, we tried to add amino acids to improve the production of EGT, but there was no apparent increase with the addition of cysteine and methionine. Besides, the addition of approximately 10 mM histidine slightly enhanced the EGT content by more than 4–5 mg/L. Considering industrial production applications, only using the basal medium was more economical. Interestingly, we found that the addition of more than 10 mM amino acids would decrease the EGT content, and over 30 mM obviously restrained the yeast growth. We suggest that the metabolism in cells is based on the carbon and nitrogen sources. Any addition of mM levels of foreign substances will change the metabolism pathway [34, 35].

**Fig. 6:**
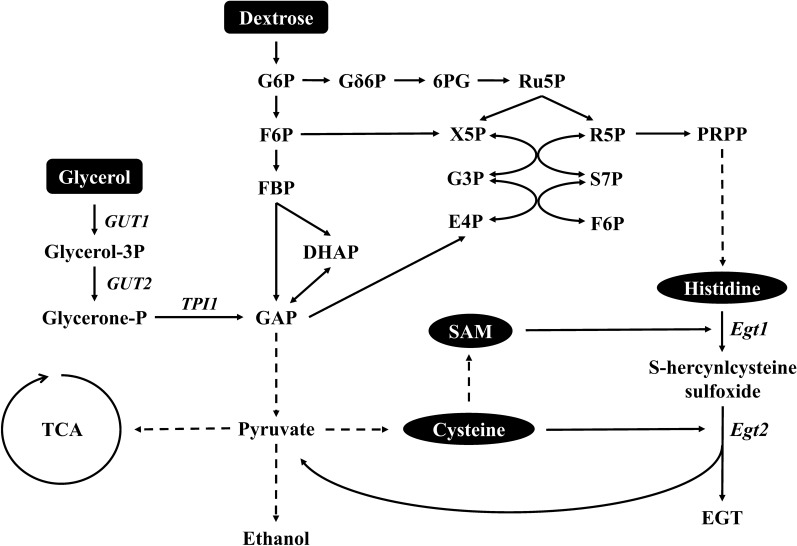
Metabolic pathways involved in EGT biosynthesis in *S. cerevisiae*

The synthetic pathway of EGT involves multiple amino acid anabolic pathways, and different carbon sources result in different contents of the reaction substrate. Glycerol, when used as a carbon source, showed benefits over dextrose in producing EGT in *S. cerevisiae*. After glycerol enters yeast cells, it is first phosphorylated by glycerol kinase (encoded by *GUT1*) to glycerol-3P, followed by the reduction of FAD-dependent glycerol 3-phosphate dehydrogenase (encoded by *GUT2*) to glycerone-P. The other predicting pathways of dissimilation are the decomposition of glycerol by the ‘DHA pathway’ and the ‘GA pathway’ [36]. The ultimate products of these three different dissimilation pathways are only glycerone-P, which is then catalysed by triosephosphate isomerase (encoded by *TPI1*) to glyceraldehyde-3P (GAP), a precursor of glycolysis and the tricarboxylic acid (TCA) cycle (Fig. 6). In contrast, if dextrose as a carbon source is assimilated by *S. cerevisiae*, its dissimilation is constructed by multiple metabolic pathways, which may reduce the synthesis effectiveness of the many important precursor substance in EGT synthesis (Fig. 6). Researches on the cultivation of yeast by glycerol have focused on the growth mechanism. Most wild-type and trial *S. cerevisiae* are difficult to grow in glycerol that is the carbon source medium [37]. Interestingly, Ho et al. [38] found that when glycerol was used as a carbon source, the strength of the promoters ALD4p and ADH2p associated with ethanol oxidation was reduced by 4 times compared with dextrose, meaning that the ethanol synthetic pathway declined and its upstream pathway obtained more sources. Besides, Strucko et al. [39] indicated that the change in the carbon source leads to the change of genome, in which the TCA cycle was decoupled from oxidative phosphorylation, thereby hampering ethanol utilization. Moreover, the change in TCA provides one NADH per C-mole when metabolized, and NADH may react with pyruvate, the accessory product from the reaction of GfEgt2, promoting the GfEgt2 catalytic rate. Specifically, we focused on cell growth after the recombinant strain was activated with YPD and fermented in glycerol or dextrose and found that yeast from glycerol medium grew slower than that grown from dextrose medium. This phenomenon was similar with diauxic shift, which reveal when fermentation yeasts reach a stationary phase after exhausting dextrose and start using by-products such as glycerol [40] as a carbon source to continue fermentation [41]. Our fermentation strategy utilized this phenomenon earlier and caused the expression of genes to change. For example, genes of energy metabolism, carbohydrate storage, gluconeogenesis, TCA and glyoxal cycles were highly upregulated [39]. When the expression levels of genes in the TCA cycle were upregulated, pyruvate, as the main source of the TCA cycle, was consumed in large quantities, thus promoting the rate of GfEgt2 enzymatic reactions and increasing the production of EGT.

## Conclusion

Our research cloned EGT synthetic genes from the mushroom *G. frondosa* and explored the possibility of expressing these genes on *S. cerevisiae*. The results revealed that both of them had a predicted enzyme activity, and we regarded glycerol as a suitable carbon source to produce EGT up to approximately 20.61 mg/L after 168 h optimized fermentation. There were only two genes we chose to construct for the EGT biosynthesis strain, which were more simplified than the five genes reported [30]. The empirical findings in this study provide a *S. cerevisiae* cell factory in producing EGT and a new understanding of EGT biosynthesis in mushroom field.

## Materials and methods

### Strains and plasmids

The mushroom *G. frondosa* was obtained from Beijing Lucky Mushroom Garden (China) and provided a source of cloning the EGT biosynthetic genes. The *Saccharomyces cerevisiae* EC1118 strain is a commercial wine yeast strain that was isolated, studied and selected from Champagne fermentations and then produced and commercialized by Lallemand (Ontario, Canada). *Escherichia coli* DH5a (Takara, Japan) was used for cloning host. The cloning vector was pMD18T (Takara, Japan) with ampicillin resistance, and the expression vectors were constructed based on the pRS42K shuttle vector with geneticin resistance, which was presented by Professor Christof Taxis and Professor Michael Knop from the European Molecular Biology Laboratory [42].

### Cloning *Gfegt1* and *Gfegt2*

The mushroom *G. frondosa* was cultured at 25 °C with 150 rpm shaking in PDB (20% potato, 2% dextrose, 0.15% MgSO_4_·7H_2_O, and 0.3% KH_2_PO_4_). Once the jar was fully colonized by the fungus ball, mycelia were collected from the liquid culture by filtering. For the extraction of fungal RNA, the mycelia were disrupted by grinding in liquid nitrogen, and then total RNA was extracted from the powder using TRNzol reagent (Tiangen, China). The TransScript Kit (TransGen, China) was used to synthesize the first-strand cDNA from total RNA of *G. frondosa*. The Kod-Fx enzyme (Toyobo, Japan) was used for the accurate PCR amplification of DNA. The cloned DNA sequences were named *Gfegt1* and *Gfegt2*. The entire *Gfegt1* open reading frame (ORF) was amplified with the forward primer, *Gfegt1*-F, 5′-atgtcgaccctccaggatttcttc-3′ and the reverse primer, *Gfegt1*-R, 5′-ttacacatcatacgcaatccgagcg-3′. The entire *Gfegt2* ORF was amplified with the forward primer, *Gfegt2*-F, 5′-atgacggccatagatctaggtgc-3′ and the reverse primer, *Gfegt2*-R, 5′-ctaattacgcttctctgcgagaagg-3′. The PCR products were purified, and an A base was added at the end of the 3′ fragment. Then, the pMD18T plasmid was connected and translated into *E. coli* DH5α for sequencing and preservation.

### Construction of recombinant *S. cerevisiae* EC1118

pRS42K was the basis of the expression vector. pRS42K was double digested with *Sal*I and *Eco*RI (Thermo Fisher Scientific, USA) and recombined with the promotor TEF1p, terminator CYC1t, *Gfegt1*, and *Gfegt2* by using the ClonExpress Ultra One Step Cloning Kit (Vazyme, China). The plasmids pRS42K (control), pRS42K-*Gfegt1* (contain *Gfegt1*), pRS42K-*Gfegt2* (contain *Gfegt2*), and pRS42K-*Gfegt1*-*Gfegt2* (contain *Gfegt1* and *Gfegt2*) (Fig. 2a) were transformed to *S. cerevisiae* EC1118 by the LiAc/SS carrier DNA/PEG method [43]. The growth of recombinant strains was tested on YPD plates (2% peptone, 2% dextrose, and 1% yeast extract) with the addition of 200 mg/L G418. The Kod-Fx enzyme (Toyobo, Japan) was used for the PCR analysis.

### Fermentation methods

Recombinant *S. cerevisiae* EC1118 was activated by YPD broths with 200 mg/L G418 and cultured overnight at 30 °C and 200 rpm. These cells were washed with ddH_2_O and then adjusted to OD_600_ = 2.0. The strains were inoculated (1% v/v) into a new fermentation medium without antibiotics for subsequent multiple fermentations. Different carbon source fermentations used YPX (Y for 1% yeast extract, P for 2% peptone, and X for 2% dextrose, 2% fructose, 2% glycerol, 2% maltose, or 2% sucrose). Different concentrations (1%, 2%, 3%, 4%, 5%, and 6%) of glycerol fermentation used YPG (Y for 1% yeast extract, P for 2% peptone, and G for glycerol). Daily supply of 1% glycerol sample means directly adding 1% glycerol (based on YPG volume) into YPG medium in each fermentation day and the control sample means only ferment in YPG (Y for 1% yeast extract, P for 2% peptone, and G for 2% glycerol) without any addition.

After fermentation, the content was centrifuged at 3000×*g* for 5 min at 25 °C to separate the culture and yeast, and then the yeast was incubated with 1 ml 50% ethanol at 50 °C for 30 min. The supernatant from the culture and cell extraction from the yeast incubation were collected and filtered through a 0.22 µm filter and then analysed by HPLC.

### HPLC analysis of ergothioneine

HPLC analyses of the above supernatants were performed on a Shimadzu LC-2030 HPLC system (Shimadzu, Japan) equipped with an Ultimate^®^ HILIC Amphion II column (4.6 mm × 150 mm, 5 μm; Welch, China) with a matching guard column. For the EGT detection, 20 μL samples were separated with 20% water/80% acetonitrile at a flow rate of 1.0 mL/min at 30 °C. EGT was detected at 254 nm at a peak retention time of approximately 21.0 min.

### UPLC-ESI–MS analysis of ergothioneine

Mass spectrometry detection was performed by a High Definition Mass Spectrometer (Synapt G2-Si, Waters, USA). The chromatographic analysis was performed in an ACQUITY UPLC System (Waters). An aliquot of 1 μL of sample solution was injected into an ACQUITY UPLC BEH Amide C18 column (2.1 mm × 100 mm, 1.7 μm; Waters) at 35 °C, and the flow rate was 0.4 mL/min. The optimal mobile phase consisted of a linear gradient system of (A) acetonitrile and (B) water, 0–4.0 min, 80% A; 4.0–4.5 min, 80–60% A; 4.5–7.0 min, 60% A; 7.0–7.2 min, 60–80% A; and 7.2–10.0 min, 80% A. The optimized analysis conditions were as follows: source temperature. 120 °C; desolvation gas temperature, 350 °C; cone gas flow, 40 L/h; desolvation gas flow, 1000 L/h; and capillary voltage, 2.3 kV.

## Supplementary information

**Additional file 1.** Additional tables.

## Data Availability

All data generated or analyzed during this study are included in this published article and the additional file.

## Abbreviations

EGT: Ergothioneine
GRAS: Generally recognized as safe
ORF: Open reading frame
SAM: *S*-adenosyl-l-methionine
PLP: Pyridoxal phosphate
TCA: Tricarboxylic acid
