# Supplementary material for: Successful biosynthesis of natural antioxidant ergothioneine in Saccharomyces cerevisiae required only two genes from Grifola frondosa
Source: Microb Cell Fact. 2020 Aug 18;19:164. doi: 10.1186/s12934-020-01421-1 (PMC7437059; doi:10.1186/s12934-020-01421-1)
Supplement: Supplementary file 1 — Additional file 1. Additional tables. [file 12934_2020_1421_MOESM1_ESM.docx]

**Additional tables**

Highly efficient biosynthesis of natural antioxidant ergotheionine in *Saccharomyces cereiviceae* required only two genes from *Grifola frondosa*

Ying-Hao Yu^1,2^, Hong-Yu Pan^1,2^, Li-Qiong Guo^1,2,^*, Jun-Fang Lin^1,2,^*, Han-Lu Liao^1^, Hao-Ying Li^1^

^1^ Department of Bioengineering, College of Food Science and Institute of Food Biotechnology, South China Agricultural University, Guangzhou 510640, China

^2^ Research Center for Micro-Ecological Agent Engineering and Technology of Guangdong Province. Guangzhou 510640, China

^*^ Corresponding author Jun-Fang Lin (J. F. Lin). E-mail address: linjf@scau.edu.cn. Tel.: +862087570302; Fax: +862085280270.

^*^ Corresponding author Li-Qiong Guo (L. Q. Guo). E-mail address: guolq@scau.edu.cn. Tel.: +862087570302; Fax: +862085280270

**Table S1.** EGT production in *S. cerevisiae* containing different construction plasmids

| **Construction types of strains** | **EGT (mg/L)** |
| --- | --- |
| WT | N.D. |
| pRS42K | N.D. |
| pRS42K-*Gfegt1* | 1.74 ± 0.02 ^b^ |
| pRS42K-*Gfegt2* | N.D. |
| pRS42K-*Gfegt1*-*Gfegt2* | 2.76 ± 0.04 ^a^ |

**Table S2.** Different carbon source for EGT biosynthesis

| **Carbon source** | **EGT (mg/L)** |
| --- | --- |
| Dextrose | 3.92 ± 0.06 ^b^ |
| Fructose | 3.86 ± 0.09 ^b^ |
| Glycerol | 13.03 ± 0.77 ^a^ |
| Maltose | 3.66 ± 0.06 ^b^ |
| Sucrose | 3.64 ± 0.16 ^b^ |

**Table S3.** Different glycerol concentration for EGT biosynthesis

| **Glycerol concentration** | **EGT (mg/L)** |
| --- | --- |
| 1% | 5.39 ± 0.08 ^b^ |
| 2% | 11.79 ± 0.77 ^a^ |
| 3% | 11.95 ± 0.24 ^a^ |
| 4% | 10.75 ± 0.54 ^a^ |
| 5% | 11.94 ± 1.33 ^a^ |
| 6% | 8.89 ± 1.23 ^ab^ |

**Table S4.** EGT contents and yeasts growth in each fermentation day

| **Carbon source** | **Day** | **OD_600_** | **EGT (mg/L)** |
| --- | --- | --- | --- |
| Dextrose | 1 | 24.18 ± 0.21 ^c^ | 1.53 ± 0.05 ^d^ |
|  | 2 | 28.25 ± 0.12 ^b^ | 2.42 ± 0.11 ^c^ |
|  | 3 | 28.81 ± 0.07 ^a^ | 2.74 ± 0.02 ^bc^ |
|  | 4 | 28.67 ± 0.09 ^ab^ | 2.84 ± 0.04 ^ab^ |
|  | 5 | 29.04 ± 0.06 ^a^ | 2.67 ± 0.07 ^bc^ |
|  | 6 | 28.68 ± 0.05 ^ab^ | 2.93 ± 0.12 ^ab^ |
|  | 7 | 28.98 ± 0.11 ^a^ | 3.18 ± 0.06 ^a^ |
|  | | | |
| Glycerol | 1 | 11.02 ± 0.18 ^c^ | 1.31 ± 0.02 ^d^ |
|  | 2 | 25.78 ± 0.21 ^b^ | 5.31 ± 0.07 ^c^ |
|  | 3 | 28.95 ± 0.24 ^a^ | 9.61 ± 0.46 ^b^ |
|  | 4 | 28.98 ± 0.40 ^a^ | 10.68 ± 0.76 ^ab^ |
|  | 5 | 29.30 ± 0.46 ^a^ | 10.64 ± 0.50 ^ab^ |
|  | 6 | 29.14 ± 0.41 ^a^ | 10.68 ± 0.31 ^ab^ |
|  | 7 | 30.26 ± 0.69 ^a^ | 11.72 ± 0.39 ^a^ |

**Table S5.** EGT biosynthesis through daily supply of glycerol

| **Treatment** | **Position** | **EGT (mg/L)** |
| --- | --- | --- |
| CK | Intracellular | 11.80 ± 0.78 ^a^ |
|  | Extracellular | N.D. |
| Treatment | Intracellular | 12.33 ± 0.65 ^a^ |
|  | Extracellular | 8.28 ± 0.80 ^b^ |

Data (Table S1 - S5) are shown as average ± standard error in triplicate tests.

Different superscript letter indicates significant difference in Tukey analysis (P > 0.05).

N.D., not detected.

**Table S6.** EGT biosynthetic genes of *Grifola frondosa*

| **Name** | **Sequence** |
| --- | --- |
| ***Gfegt1*** | ATGTCGACCCTCCAGGATTTCTTCCATATCGTGGACCTTCGTGCCAACCAGCCAACACTTGCTTCCAGCGTCATCCATGAACAAGTTGTTTCCGGTCTCTCGCAACCTGCGGGCCAGAAATGGCTTCCCACAATGCTCCTCTACGATGAGAGGGGATTGAGGCTGTACGATGCCATTACGACAGAGGCGCCCGAATACTATTTGTTTCCCGCCGAGGAAGAGATCCTGAAGAACCGGTCTTCCGATATTGTGCGGGTCATGCATGCGCGGAACGGGAATGCAGAGTCAGTTGAAGAGGTCGTCGTTGAGCTTGGTGCTGGTGCTTTAAGGAAGACATCCCACATCCTCCGCGCTCTCTCACAGCACAGTATGTCTTCCGTCCAGTACTACGCCCTCGACCTTGAGAAGCGCGAACTCGAACGCACTCTCAAGACACTACATGACTCTGAAATTGGAGCAGAGATCAAGGATAAGGTCTCTACTAAGGGCTTGTGCGGAACATATGACGACGGCCTCAAGTTCATCGCCGAGGGTGGCCTGGAAGGACGCAACGATCTTGAACGGATCACTACTGAAGTCTCCGAGCAATATAAGCTCGAGAGGGTTGGCGGTGACGATTCGCCTAGATCTGCGTCTTCCTCGAGGACACCTACGACGGAGACAGATGTCACACCTCCATCGACCCCTGGTTTTAACCAGCCGCTTCATATTCTCTTCCTCGGTTCATCGCTCGGCAACTTCACTCGTGGTGAGGATGCTGCATTCTTACGATCCTTGCCGTTGCGACCTGGTTCAGGCGATACATTGCTCCTGGGCCTCGACCACGACAATGAAGCCCATCAGATTGAGCTCGCATATAATGACCCCAAAGGTATCACCAAGAATTTCATTATGAACGGCTTGAAATGTGCAGGAAGAGCTCTTGGGGACGAGCACCTCTTTGATGAAGATAAATGGGAGTATGTCGCGATGTACAACGAAGAACTCCGTCGTCATGAGGCTTACTACAAGTCAACGTGTGAGCAAACGGTTGTGGACACAAAGACTAAGAAGTGTCTCCCGTTCGAAGCAGACGAGCTCGTCCGCATCGAGGTTTCCTACAAGTTCTCTGAGCGAGACGCGTACACTCTTTTTACTGACGCCAATCTCCGCCCCATTCAACGCTGGATGGACAGCGCTGGGCAGTATTCTCTCTGGCTGCTAGAGCGACCTAAGTTCACGTTCCCTCTACTGCGCTCGCCTTCCGCCATTGATGAGAAGGGTGTGGTTTCTTCTCCTTTCGGCATGCCAGCAATGGACGAGTGGCACACAATGTGGGCGGCTTGGGACTTTATCACTAGACAGATGATACCCCCTTCGATGCTCTTCCAGAAGCCGATCGATCTACGTCATATATGCCTGTTCTATTGCGGGCATATTCCTGCGTTCCTGTCCATTCATATTTCGAAGCTTCTTGAGGAGCCGGACACCGAACCTGTGGAGTTCAAATATATTTTTGAACGAGGGATCGATCCAATTGTGGATGACCCCACCAAGTGCCACCCTCACTCTGAGGTTCCGCAGCATGACGAGGATTGGCCTTCGCTCGGGAGCATTCTTGAATACCAATCTAGGGTGCGCGAGCGAGTGATGAAATTATACCGCGATATCCAGTCTGGGAAAGTTACGCTCACGAGGAAGATAGCCAGAGTCTTGTTTATGACACTCGAACACGAAGCTTTCCATGCTGAGACACTCTTGTATATGTTGTTGCAGCGCGCGGGCACGGGCACATTGCCTCCTACGGGCTTCAGTCCGCCAGTCTGGTCTGTCCTCGCCGAGTCTTGGGAACGCCTCCCTGCTCCGCATACTCCCACCGTGACGCTCGGTCCGGAGACACTGACGGTCGGACATGATGACAGCGAAGCGGATGACAATACCACCGACGTGGCTGGACATGAGTTTGGCTGGGACAACGAGCACCCCAAAAGGACCGTGCATGTTCCGGAATTCAAGATCGAGTGGCGCCCTGTTACGAACGGAGAGTTCTACGAGTTCTACATTGGAGAAGGCAAGGAACAAGTGCAGTTGCCTGCCAGCTGGGTGGAGATCGACGGGGAGATGCTGGTGCGCACCTTCTACGGACCAGTCCCGATGAAAGTGGCAAAAGACTGGCCGGTCATCACGTCCTACGATAATCTCTCTACCTACGCCAGCGTCAAGGGCGGCCGCATCCCCACCGAGCCTGAACTCCGCTTGTTCCTCGACAAGTTCGAGTGCGGATACGAAGGCGGGGCAAATATTGGCTTCCGCAACTGGCACCCTATTCCGGCGACTATGGGCGGGGTGAAAGATGGCCGGGGACACAACGGAGGTGTCTGGGAGTGGACGTCGACGGTGTTCGAGAAACATGACGGTTTTGTGCCGTCCAAGCTGTATCCGGGATATTCGATGGATTTCTTCGATACCCACCATCAAATTGTGATCGGAGGCTCCTACGCCACTATTCCCCGTCTCGCGGAGCGGCGTACCTTGCGTAACTACTACCAACACAACTACCCCTACGCGTGGGTCGGCGCTCGGATTGCGTATGATGTGTAA |
| ***Gfegt2*** | ATGACGGCCATAGATCTAGGTGCAGCGTCTGCCGACGAGACCCAGAAGAACACTTATGATGCAACCCAAAAGCCGCCTCCTTTCGGTCATGCCTTGAAGCCTTACTGGGCATTTGATCCAAAATATGTAAATCTAAACCACGGCTCCTATGGATCATTGCCTCTGCCTGTTCTATTTTCTTGCACCCAGAATACGATTCTCGCAGAGCGGAATCCCGACAAATTCCACCGCGTCACGTATATGCCTATGCTCCAGGAGTCCAGGAAACGCGTGGCAGAACTGGTTGGTGCTGAACACGACGAGATCGTGCTCGTGCCTAATGCCACTCACGGCTTGAACACCGTGCTCAGAAATTTTGAGTGGAAGCAAGGCGACGTCATTATTGGAGCATCGACGACATATGGTGCCATCTCTCGCACCATCCAATACCTCGCGGATCGATCGGAACAGCCAAGACCCGAAGCATATAGCATTCAGTATACGTTCCCCATGTCGCACGCAGAGATCCTCGATGCCTTCCGTGCACGCGTGCGGGAGATCAAGCAGCTCCATGCGAGCACCGAATTCAGCGACGCGCCGTTGGAGTCGCTGGGCTACGAGGAAGGCAGGAAGAAGAACAAGTTCGTCGCAGTTATAGACTCGGTGACTGCCAACCCTGGGGTCCTCATGCCCTGGAAAGAGATGGTCCGCGTCTGCAGGGAAGAAGGCATCTGGTCTGTTGTAGATGCTGCTCATAGCATCGGGCAGGAAACGGATATCAATCTCAGCGAAGCGAGGCCTGATTTCTGGATATCCAACTGTCATAAGTGGCTTTACGCAAAACGGGGCTGTGCCACCTTATATGTGCCCAAACGTAACCAGTATATCATCAAGTCTTCTATTCCGACTTCACACGCGTATGTCTCGCCTACCGACACAGAACAGGCCCTGCAATTCCGGGATGGGTATGACACGAATTTCATCCTGCAGCATGAATGGACAGGGACGATGGACTTCATTCCATACTTGAGCGTCTCTGCAGCGCTCGACTTCCGCAACTGGCTGGGAGGGGAGGCCGCCATCAACGGGTACTGCCACAAGCTCGCCATGGCCGGCGGCGAGAGGCTCGCCAGCGTGATGGGCACGAAGGTCATGGACAAGACCGGCGAGCTCACGCTCAACATGACGAACGTCCTACTGCCGCTCCCCGTGGAGACTACGAAGGGCGAGGTGTATTCTGGGGAGGTCCTGTCCGCGATTTACAGCCAACTCAGGGAGAAGCTGCTGTACGAGTGGAACACCTACGCGGCACACTACTTCCACGCGGGCGGCTGGTGGTGCCGGTGCAGCGCACAGGTCTGGAACGAGGAATCGGACTTTGAGTATCTGGGAAAGGCATTCAATGCAATCTGCAAGGAAATCAAGGATACCCTTCTCGCAGAGAAGCGTAATTAG |

|  |  |
| --- | --- |
